# Editorial: Superheroes and villains: engagement, effects, and empowerment

**DOI:** 10.3389/fpsyg.2025.1720266

**Published:** 2025-11-03

**Authors:** Julia Kneer, Daniel Pietschmann, Bartosz Grzegorz Żerebecki

**Affiliations:** ^1^Media and Communication, Erasmus School of History, Culture and Communication, Erasmus University Rotterdam, Rotterdam, Netherlands; ^2^Faculty of Humanities, Institute for Media Research, Chemnitz University of Technology, Chemnitz, Germany

**Keywords:** superheroes, villains, character engagement, parasocial relationship, role model, education

## Introduction

While many of the modern superheroes took firm roots in media around the late 1930s (Packer, 2009), the stories of extraordinary trials and tribulations against villains have been circulating for much longer. Some even argue the genre emerged due to its evolutionary advantage, namely, that superheroes may help to enforce prosocial norms within large groups (Carney et al., 2014). Today, engagement with characters of supernatural abilities is no longer restricted to reading books or graphic novels. Superheroes and superheroines of various backgrounds, races, gender identities, and sexual orientations increasingly feature in global cinema and streaming content. Moreover, they have found their way into classrooms and therapeutic settings (Scarlet, 2016).

This sharp rise in popularity across diverse domains demonstrates that the narratives of the brave and the fallen provide far more than mere entertainment. In fact, they shape how individuals understand and negotiate questions of morality, resilience, identity, and empowerment. In this editorial, part of the Research Topic: *Superheroes and villains: engagement, effects, and empowerment*, we discuss the diverse contributions that collectively illustrate the psychological significance carry for their audiences.

## Superheroes and villains as (anti) role models?

Superheroes can serve as role models for the youngest media consumers. Think of children pretending to be their favorite characters. But is this engagement innocuous or does it have serious consequences? Questions about whether role-playing fosters moral development, or potentially hinders it through stereotyping or promoting aggressive behavior, remain relevant.

The article *Mini marvels: superhero engagement across early childhood* indicates that even the youngest audiences actively engage with superhero characters through role-play and identification (Coyne et al.). While most forms of superhero engagement had no impact on later behavior, high early identification and toy use were found to predict future aggressive and defending behaviors. These findings serve as a reminder that superheroes are not merely sources of fun; they also play a role in the behavioral development.

Given that superheroes may shape children's development, could they also serve as tools in therapy. The case study *Regression in the service of bibliotherapy—What can “Captain Underpants” teach us?* showcases how superhero narratives can aid therapeutic work (Ifrah). The author describes her work with David, a child with learning disabilities, who used Captain Underpants to playfully engage with social taboos such as bodily excretions. Creating his own storylines helped David express emotional states, with identification with a self-created villainous superhero serving a therapeutic role. Reflecting on fictional characters, be it villainous or heroic, and linking them to real-life experiences can be profoundly empowering.

Educators are undoubtedly champions of children's growth, but can superhero narratives also offer insights for teaching? The opinion piece *Master Splinter and the challenge of personalization* examines different forms of personalized education (Triberti and Di Fuccio). The authors argue that Master Splinter's teaching style invites more active and counterintuitive forms of personalization than commonly assumed by contemporary educators; for instance training each turtle with a weapons that challenges, rather than aligns with, their personalities. This piece adds an innovative perspective to discussions of educational differentiation and student development.

## Superheroes vs. villains—or a joint adventure?

While the first set of contributions focuses on how superheroes function in early development and education, the second set turns to the complex psychological appeal of both heroes and villains in later life and media engagement.

Early comic books often drew a stark line between good and evil. Yet, heroes and villains may have more in common than expected. While villainyis often rooted in suffering and pain, heroes also possess vulnerabilities and may cross into moral gray zones. Recognizing positive and negative traits in both heroic and villainous characters deepens audiences engagement and emotional resonance.

This complexity is addressed in the article *Faces of depression. Why do we need Batman, Joker, and Bane?* (Reghintovschi and Reghintovschi). The authors argue that Christopher Nolan's Batman Trilogy mirrors depressive symptoms that many viewers may experience. Characters like Batman, Joker, and Bane represent internal conflicts related to grief and guilt. Through identification with these symbolic “masks,” viewers may externalize their own struggles, gaining psychological distance, and potentially, hope for healing.

A similar interplay of moral ambiguity emerges in *The attraction of evil. An investigation of factors explaining women's romantic parasocial relationships with bad guys in movies and series* (Schramm and Sartorius). The authors ask why some women are drawn to “bad boy” characters and explore the development of romantic parasocial relationships (RPSRs) with them. Results show that a playful, non-committal love style and a high level of sensation seeking are associated with stronger RPSRs. Morally ambiguous characters allow audiences to safely engage with their own “darker sides” and reflect on identity, emotions, and connections. Sometimes, it takes both good and evil for growth to occur.

## Conclusion

The five contributions in this Research Topic reflect a diverse range of methods (longitudinal research, case study, conceptual analysis, and opinion pieces) underscoring the richness and complexity of this area. Together, they attest to the many roles and meanings superheroes (and their villains) carry in the contemporary world.

These stories of extraordinary deeds influence children, therapy patients, educators, and general audiences alike. Future research could further explore how superhero narratives function across cultures, life stages, and media formats, or how individuals internalize and reframe moral ambiguity over time. We hope that this Research Topic encourages continued inquriy into how superhero narratives help people understand themselves and others, while offering pathways for resilience, reflection, and connection.
